# Nanoparticle Delivery of Antisense miR162 Inhibits Invasive Habitat Adaption of *Alternanthera Philoxeroides*

**DOI:** 10.1002/advs.202416747

**Published:** 2025-03-31

**Authors:** Qianqian Hu, Erfeng Kou, Xiuzhen Liao, Ruiyi Qiu, Qi Tang, Huan Zhang, Yun Zheng, Ji Yang, Binglian Zheng

**Affiliations:** ^1^ State Key Laboratory of Genetic Engineering Ministry of Education Key Laboratory of Biodiversity Sciences and Ecological Engineering Institute of Plant Biology School of Life Sciences Fudan University Shanghai 200438 China; ^2^ School of Agriculture and Biology Shanghai Jiao Tong University Shanghai 200240 China; ^3^ College of Landscape and Horticulture Yunnan Agricultural University No. 95 Jinhei Road Yunnan 650201 China; ^4^ Ministry of Education Key Laboratory for Biodiversity Science and Ecological Engineering Institute of Biodiversity Science Fudan University Shanghai 200438 China

**Keywords:** *Alternanthera philoxeroides*, invasive plants, miRNA, phenotypic plasticity

## Abstract

Phenotypic flexibility in adaptive traits is crucial for organisms to thrive in changing environments. ***Alternanthera philoxeroides***, native to South America, has become an invasive weed in Asia. The mechanism by which invasive capacity is achieved remains unknown. Here, it is demonstrated that miR162 plays a crucial role in submergence survival **for *A. philoxeroides***. These results highlight that the level of miR162 significantly increases in stems from 3 to 48 h upon water submergence, and knockdown of miR162 via TRV‐based VIGS system significantly disrupts stem elongation upon water submergence, ultimately resulting in a failure of plants protruding from the water surface. Interestingly, miR162 is not up‐regulated in the noninvasive congeneric alien species ***Alternanthera pungens***, which is also native to South America but has retained its original habitats in Asia. The presence of anaerobic responsive elements (AREs) in the promoter sequences of *MIR162* from ***A. philoxeroides*** rather than ***A. pungens*** may contribute to its invasion capacity. Importantly, nanoparticle delivery of antisense RNA oligonucleotides of miR162 significantly impairs stem elongation during water submergence. Thus, our findings reveal that the achievement of specific miRNA activity can drive rapid phenotypic variation, and miR162 has the potential as a bio‐pesticide for controlling the invasive growth of ***A. philoxeroides***.

## Graphical abstract

This study demonstrates that the miRNA pathway plays a key role in the invasive growth of *Alternanthera philoxeroides* in aquatic habitats. Specifically, miR162 is critical for its invasive success, facilitating stem elongation and enhancing survival during submergence. Nanoparticle‐mediated delivery of antisense RNA oligonucleotides targeting miR162 effectively suppressed stem elongation, underscoring its potential as a biopesticide to control this invasive weed.

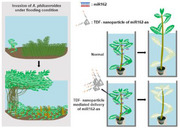

## 1 Introduction

The invasive plant *Alternanthera philoxeroides*, commonly known as alligator weed, has emerged as a significant ecological threat in various environments. Native to South America, this species has rapidly spread to diverse regions across the globe, including North America, Asia, and Australia, due to its high reproductive potential and adaptability.^[^ ^1^ ^]^ The absence of its native predator, *Agasicles hygrophila*, and its robust clonal propagation system, characterized by its ability to produce adventitious roots and shoots from stem fragments, facilitates rapid and extensive colonization.^[^ ^2^ ^]^ *A. philoxeroides* forms dense mats on the water's surface, leading to profound ecological disruptions. These mats block sunlight, impairing the photosynthesis of submerged aquatic plants and disrupting aquatic ecosystems. The resulting loss of plant biodiversity can lead to decreased oxygen levels in the water, negatively affecting fish and invertebrate populations. Additionally, the extensive growth of *A. philoxeroides* can hinder water flow and recreational activities, and its persistence in various habitats underscores the need for effective management strategies.^[^ ^2^, ^3^ ^]^

*A. philoxeroides* exhibits several remarkable adaptive mechanisms that contribute to its success as an invasive species. One key adaptation is its high tolerance to aquatic habitats, allowing it to rapidly grow out of the water by significantly increasing its stem internode length within a few days.^[^ ^1^ ^]^ RNA sequencing analyses have shown that the expression of numerous hormone‐responsive, stress‐responsive, and epigenetic regulatory genes is dynamically regulated in response to water submergence.^[^ ^4^, ^5^ ^]^ Although this rapid phenotypic flexibility enables *A. philoxeroides* to adapt locally, the specific mechanisms underlying this adaptive capacity remain largely unknown. Moreover, no specific factors have been identified that promote its high tolerance to aquatic environments.

miRNAs modulate gene expression by targeting specific mRNAs for degradation or translational repression, enabling the plant to fine‐tune its response to environmental changes.^[^ ^6^ ^]^ Our previous finding has identified some water submergence‐responsive miRNAs, and co‐expression network analyses show that those submergence‐responsive miRNAs may target genes involved in gibberellic acid (GA) synthesis and abscisic acid (ABA) signaling.^[^ ^5^ ^]^ These results indicate that miRNA molecules are actively involved in its high tolerance to aquatic habitats in *A. philoxeroides*. Despite these clues of miRNA dynamics on its phenotypic flexibility, whether and by which specific miRNA can contribute to the process of rapid adaptation is largely unknown.

A long‐standing question in invasive biology is how the acquisition of invasive competence is related to the land‐to‐water environmental transition. We approached this question by re‐examining the spatiotemporal expression patterns of miRNAs and the phenotypes resulting from the knockdown of the miRNA pathway. Our findings demonstrate that the miRNA pathway plays a crucial role in stem elongation under water submergence in *A. philoxeroides*. Furthermore, by characterizing the rapidly responsive miRNome of both young and old stem tissues subjected to submergence for 3 h to 4 days, we identified a specific microRNA, Aph‐miR162, that was significantly upregulated in both young and old stems from 3 to 48 h after water submergence. As expected, the knockdown of Aph‐miR162 via the TRV‐based VIGS (virus‐induced gene silencing) system significantly inhibited stem elongation in *A. philoxeroides* underwater submergence conditions, eventually resulting in plant death. Consistent with the noninvasive nature of *Alternanthera pungens*, a congeneric alien species of *A. philoxeroides*, the upregulation of miR162 in response to water submergence was not observed in *A. pungens*. Sequence analyses of the promoters of *MIR162* genes uncover that the higher presence of AREs (Anaerobic Responsive Elements) in *A. philoxeroides* compared to *A. pungens* suggests that the induction of miR162 in response to submergence is correlated with the invasive capacity of *Alternanther*. Importantly, the nanoparticle‐delivery of antisense oligonucleotides of miR162 inhibits stem elongation of *A. philoxeroides* during submergence, providing a clue for developing an RNA‐based pesticide for the control of invasive plants.

## 2 Results

### 2.1 Spatiotemporal miRNome Responsive to Aquatic Habitats in *A. philoxeroides*

**Figure 1:**
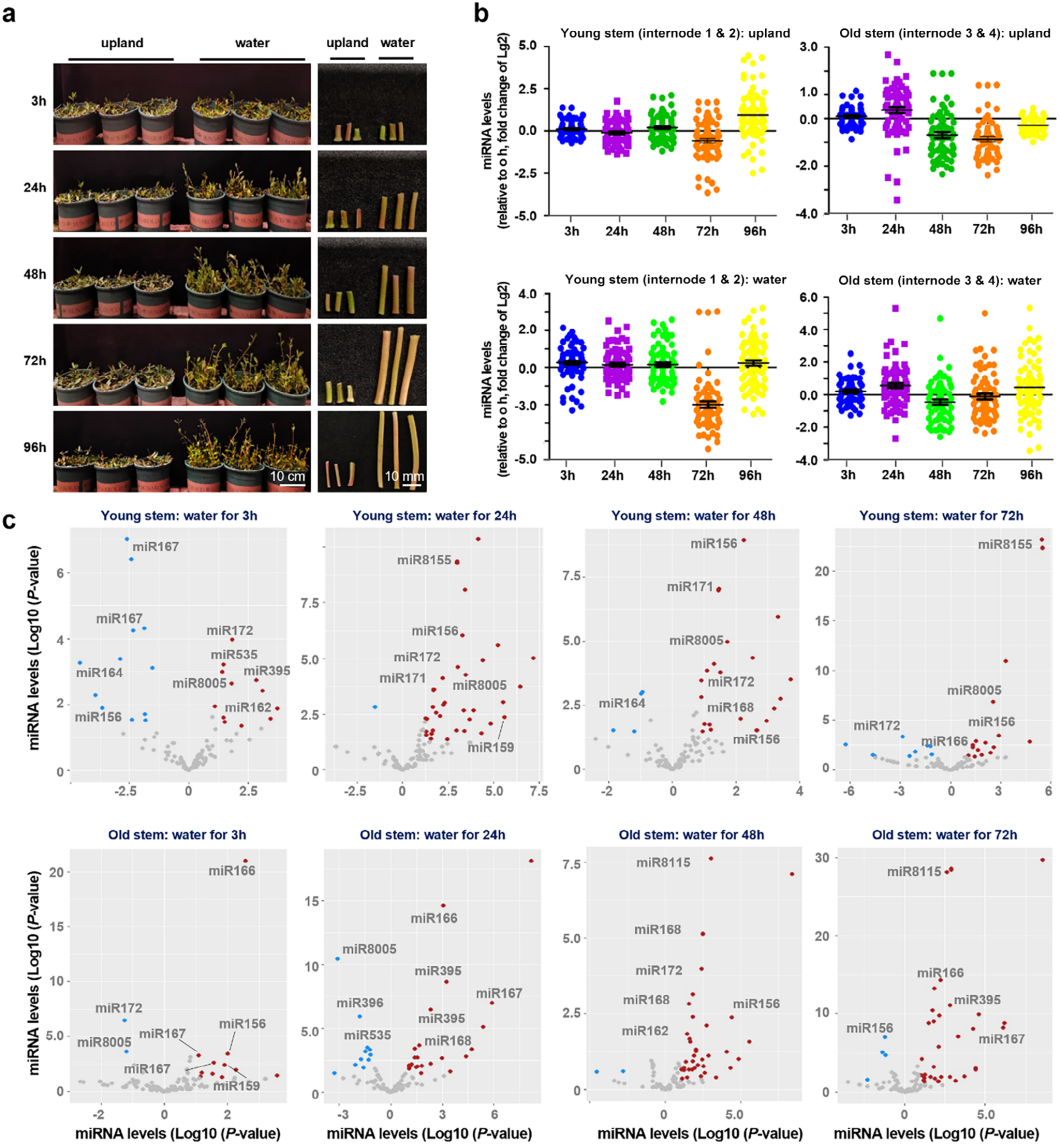
miRNA profiling responding to water submergence in *A. philoxeroides*. a) The phenotypes of upland and water‐logged *A. philoxeroides* at different time points. The left side displays the overall growth phenotypes, while the right side shows the morphology of the second internode. Scale bars are shown. b) Overall levels of miRNAs in young stems (internodes 1 & 2) and old stems (internodes 3 & 4) under different conditions. The fold changes of miRNA levels were calculated relative to 0 h. Data are presented as mean ± standard errors (SE). c) Volcano analysis showing typical differentially expressed miRNAs in stems at different time points after submergence. The red dots represent upregulation, while the blue dots represent downregulation, and the gray dots indicate no change.

Known for its remarkable adaptability, *A. philoxeroides* exhibits rapid stem elongation in response to flooding conditions, enabling it to elevate above the water surface and effectively mitigate submergence stress. To explore the role of miRNAs in this stem elongation response and their potential to regulate the plant's thriving growth in aquatic habitats, we extracted total RNA from young stems (including internodes 1 and 2) and old stems (including internodes 3 and 4) subjected to varying durations of water submergence (0, 3, 24, 48, 72, and 96 h) (**Figure** **1**a). Subsequently, we conducted comprehensive miRNA sequencing analyses. The miRNA‐seq profiles were consistent across multiple samples for both young stems and old stems at different time points (Figure S1a, Supporting Information). Then, we evaluated the global profiles of all identified miRNAs for each sample, and the results show that the overall dynamics of miRNA changes in both young and old stems were similar between upland and water submergence conditions (Figure 1b, Table S1, Supporting Information). In old stem tissues, compared to the levels observed at 0 h, *A. philoxeroides* showed a slight increase in miRNA levels at the 24 h mark under both upland and water submergence conditions (Figure 1b). However, there was a significant decrease in miRNA levels at 48 and 72 h points (Figure 1b). In contrast, the miRNA levels in young stem tissues remained relatively stable under both upland and water submergence conditions up to the 48 h mark (Figure 1b). A significant decrease was observed at 72 h, but the levels rapidly returned to normal at 96 h (Figure 1b). These analyses indicate that the overall patterns of miRNA dynamics in stem growth are comparable between upland and water submergence conditions.

Despite the general trends indicating that both growth environments influence miRNA expression in a similar manner, many specific miRNAs were found to be up‐regulated more than twofold in water submergence conditions compared to upland conditions (Figure 1b). To characterize specific miRNAs that respond to water submergence in *A. philoxeroides*, we focused on samples collected from three to 72 h of submersion. As shown in Figure 1c, several highly conserved miRNAs, including miR156, miR159, miR160, miR162, miR164, miR166, miR167, miR168, miR171, miR172, miR319, miR390, miR395, and miR396, exhibited significant de‐regulation under submerged conditions. Additionally, several non‐conserved miRNAs, such as miR535, miR8005, and miR8155, were also de‐regulated in response to water submergence (Figure 1c). This suggests that specific miRNAs may play a crucial role in the adaptive responses of *A. philoxeroides* to aquatic environments. To assess the broader significance of certain miRNA responsiveness as a common regulatory mechanism during water stress and subsequent adaptation, we examined the petiole elongation capacity of several *Arabidopsis* mutants deficient in miRNA biogenesis and activity. This phenotypic trait is crucial, as *Arabidopsis* plants typically elongate their petioles to rise above water. The results show that compared to wild type (Col‐0), *hyl1‐2* and *ago1‐27* mutants, which are known to impair miRNA processing and function, displayed significantly reduced petiole elongation after submergence (Figure S1b,e, Supporting Information). This finding underscores that miRNA plays a conserved role in facilitating rapid growth above the waterline, extending from *Arabidopsis* to Alternanthera.

Further investigations were conducted on the growth phenotypes of two specific miRNA mutants: *mir159abc* and *mir164abc*, each expected to elucidate individual miRNA contributions to submergence adaptation. Notably, *mir159abc* mutant showed a marked inhibition of petiole elongation upon submergence, suggesting its essential role in facilitating growth under these conditions. In contrast, Col‐0 and *mir164abc* mutant did not exhibit this inhibitory phenotype (Figure S1c,e, Supporting Information). Given that *MYB33* and *MYB65* are targets of miR159, we further show that *myb33 myb65* double mutant^[^ ^7^ ^]^ exhibited increased petiole elongation, contrasting with the reduced elongation seen in *mir159abc* mutant (Figure S1d,e, Supporting Information). This observation implies that the regulation of petiole elongation by miR159 may operate through *MYB33* and *MYB65*, and the miRNA‐mediated regulatory mechanisms underlying the rapid emergence of plants above the water surface under flooding conditions may be evolutionarily conserved from *Arabidopsis* to *Alternanthera*.

### 2.2 Both miRNA Biogenesis and Action Promote Stem Elongation of *A. philoxeroides* in Aquatic Habitats

**Figure 2:**
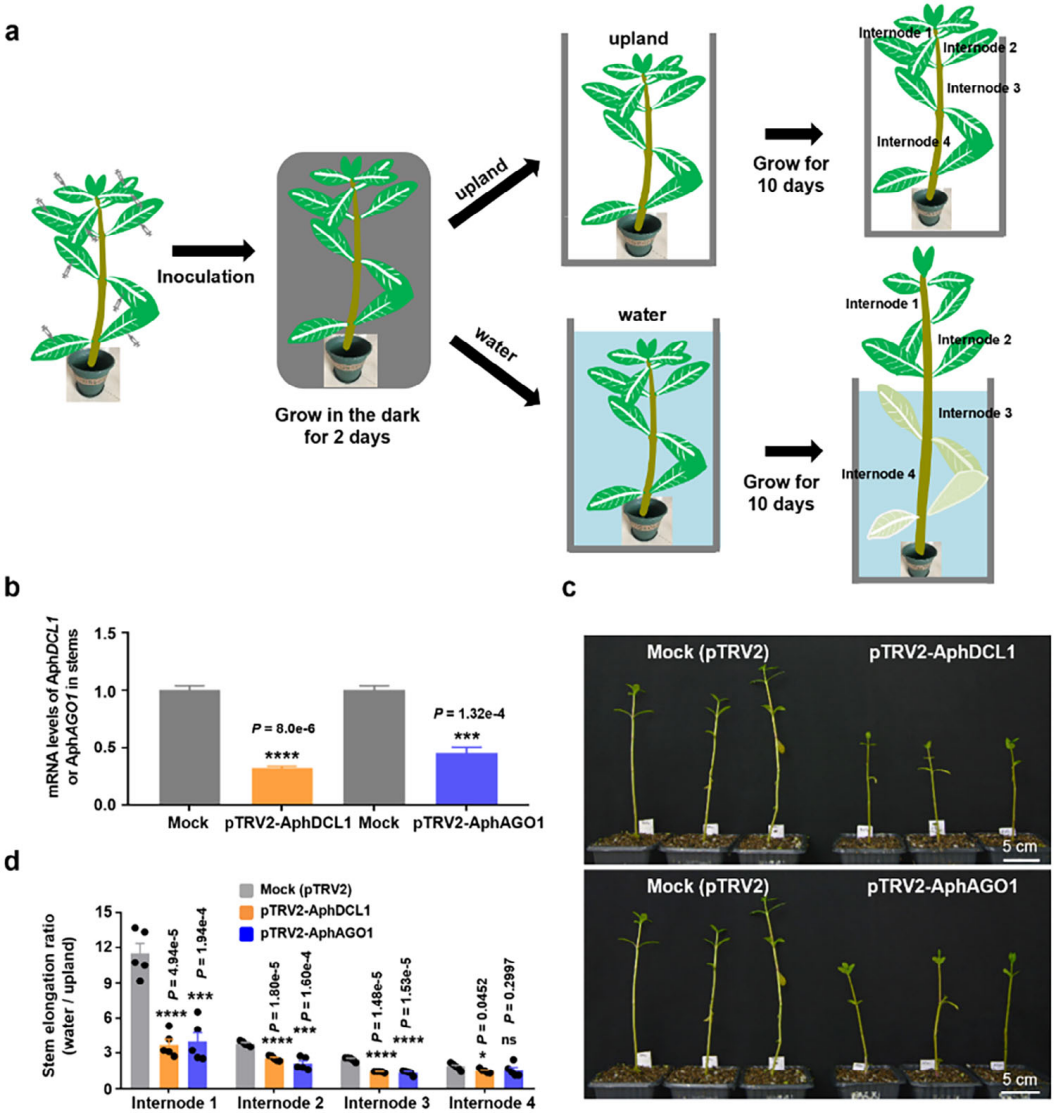
Knockdown of *AphDCL1* and *AphAGO1* impairs submergence‐induced stem elongation in *A. philoxeroides*. a) Workflow for VIGS in *A. philoxeroides*. Select uniform‐sized plants containing four internodes, and transplant them into soil, ensuring that two internodes are buried underground and two internodes remain above ground. Allow the above‐ground portion to grow two new internodes, typically containing eight to ten leaves. Next, inoculate each leaf with Agrobacterium solution containing the VIGS vectors. Keep the plants in a dark and moist environment for two days. After this period, divide the plants into two groups: one group is submerged in water, while the other group continues to grow on upland. Finally, observe the growth phenotypes after ten days. b) Expression of *AphDCL1* and *AphAGO1* in stems of VIGS‐treated plants. Data are presented as mean ± standard errors (SE) from three biological replicates. *AphUBC* was used as an internal control. c) Plant height comparison of water‐logged *A. philoxeroides* after silencing *AphDCL1* and *AphAGO1* with Mock. Scale bars = 5 cm. d) Statistical analyses of the elongation ratio of internode after submergence treatment. Five plants were analyzed for each group, and each dot represents one internode. ****p < 0.0001, ***p < 0.001, and *p < 0.05, ns indicates not significant; Student's *t*‐test was performed for (b) and (d).

Both our comprehensive miRNA profiling analyses and a previously published study have consistently demonstrated that ≈100 miRNAs exhibit significant expression changes in response to water submergence in *A. philoxeroides*,^[^ ^5^ ^]^ indicating that the entire pathway involving miRNA biogenesis and action may be necessary for adaptation to aquatic habitats. To test this hypothesis, we first needed to develop an efficient method to genetically interfere with the activity of the miRNA pathway in *A. philoxeroides*. A previous study reported that the potato virus X‐based virus‐induced gene silencing (VIGS) approach is a feasible method for knockdown of gene expression in *A. philoxeroides*.^[^ ^8^ ^]^ However, the efficiency of this method in our lab, as well as in several other labs, has been very low. This may be due to the strong resistance exhibited by the invasive plant *A. philoxeroides* to potato virus X‐based inoculation under our growth conditions. To enhance the inoculation efficiency, we focused on the TRV (tobacco rattle virus)‐based VIGS system, which has proven to be the most effective method of gene silencing in many horticultural plants and crops.^[^ ^9^, ^10^, ^11^ ^]^ TRV offers several distinct advantages over other viruses developed for VIGS, including relatively mild infection symptoms, the ability to infect large patches of neighboring cells, and efficient migration to growing meristems, thereby reaching new tissues throughout the plant.^[^ ^12^, ^13^ ^]^ Before optimizing this method, we established a workflow to assess the aquatic habitat adaptation ability of *A. philoxeroides* (**Figure** **2**a). Initial branches with four to five pairs of leaves were used for inoculation, followed by culturing the inoculated branches in darkness for two days. These branches were then split into two groups for a 10‐day growth period: one group was grown upland, while the other was submerged in water. Finally, the length of each stem segment between nodes was measured. To determine the optimal concentration of agrobacteria for efficient inoculation, we first tested two positive reporters: pTRV2‐GFP and pTRV2‐AphPDS (phytoene desaturase). Unlike the usual concentration of OD_600_ = 0.15 used for most VIGS vectors, a significantly higher concentration of OD_600_ = 0.5 of Agrobacterium strain GV3101 was required for the pTRV2 vector to inoculate the leaves of *A. philoxeroides* effectively (Figure S2a, Supporting Information). Using this optimized method, we successfully expressed GFP in *A. philoxeroides*, as evidenced by the distinct fluorescence signals detected not only within the cytoplasm of directly infiltrated leaves but also in adjacent newly developed leaves (Figure S2b, Supporting Information). Additionally, we successfully knocked down *PDS* of *A. philoxeroides*, in which the leaves inoculated with pTRV2‐AphPDS, rather than the blank pTRV vector, turned yellow, and new leaves developed a pale appearance (Figure S2c, Supporting Information). The expression level of *AphPDS* decreased to ≈30% following pTRV2‐AphPDS inoculation (Figure S2d, Supporting Information). These attempts demonstrated that TRV‐based VIGS can be used for gene silencing in *A. philoxeroides*.

To investigate whether the intact miRNA pathway is involved in the aquatic habitat adaption of *A. philoxeroides*, we employed the TRV‐based VIGS system to knock down the expression of *AphDCL1*, the main miRNA biogenesis gene, and *AphAGO1*, the main miRNA effector. The inoculation of TRV‐AphDCL1 and TRV‐AphAGO1 into the leaves resulted in the expression of the two target genes decreasing to ≈20% to 40% of that observed with the blank vector inoculation (Mock control) (Figure 2b). As shown in Figure 2c, the height of the plants significantly decreased in both cases of knocking down either *AphDCL1* or *AphAGO1*. Statistical analysis of the stem internode length from the indicated samples further confirmed that overall elongation was significantly impaired when either *AphDCL1* or *AphAGO1* was knocked down (Figure 2d). Notably, the elongation of internodes 1 and 2 was particularly affected (Figure 2d), suggesting that responsive signals are preferentially distributed at the top. Consistently, the impaired elongation of internodes 1 and 2 predominantly contributed to the observed phenotypes in both cases of *AphDCL1* and *AphAGO1* knockdown (Figure 2d). These results indicate that an intact miRNA pathway is necessary for the aquatic habitat adaptation of *A. philoxeroides*.

### 2.3 Submergence‐Induced miR162 Mediates Stem Elongation in *A. philoxeroides*

To streamline the potential development of miRNA‐based pesticides, we hypothesized that specific miRNAs might regulate stem elongation under flooding conditions. To test this, we analyzed the abundance of miRNA isoforms in *A. philoxeroides*. Based on this analysis, miR162 was selected for further study because it was significantly upregulated in the stems of *A. philoxeroides* after 3 h of flooding treatment (Figure S3a, Supporting Information). Additionally, miR162 is highly conserved across a wide range of plant species, highlighting its biological significance.^[^ ^14^ ^]^ Furthermore, miR162 is encoded by only two genes in *A. philoxeroides*, which simplifies its manipulation in knockdown experiments and ensures more effective suppression of its expression. These characteristics make miR162 an ideal candidate for developing strategies to control the invasive growth of *A. philoxeroides*.

**Figure 3:**
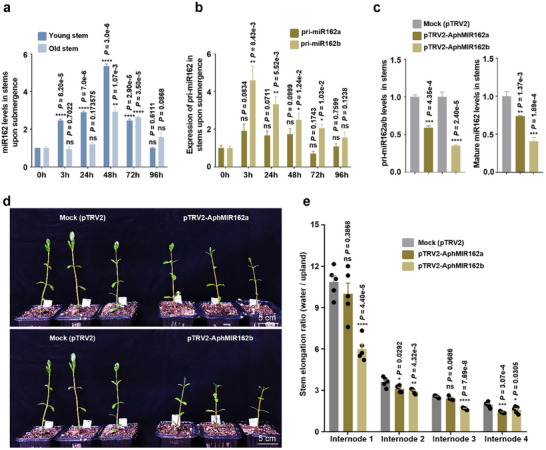
miR162 mediates submergence‐induced stem elongation in *A. philoxeroides*. a,b) RT‐qPCR analyses showing levels of miR162 (a) and pri‐miR162 (b) in stems of *A. philoxeroides* at different time points after water‐logging. c) RT‐qPCR analyses showing the levels of pre‐miR162 (left) and miR162 (right) in stems of *A. philoxeroides* inoculated by the pTRV2‐VIGS vectors. For (a–c), data are presented as mean ± standard errors (SE) from three biological replicates. AphU6 was used as an internal control. d) Growth phenotypes of water‐logged *A. philoxeroides* after silencing two *AphMIR162* genes. Mock was the control. Scale bars = 5 cm. e) Statistical analyses of the elongation ratio of internode after submergence treatment. Five plants were analyzed for each group, and each dot represents one internode. ****p < 0.0001, ***p < 0.001, **p < 0.01, and *p < 0.05, ns indicates not significant; Student's *t*‐test was performed for (a, b, c, e).

By carefully examining the dynamics of miR162 at different time points in both young stems and old stems under water submergence using RT‐ qPCR analyses, we show that in young stems, miR162 levels were rapidly increased to more than twofold at 3 h after water submergence, and this increase lasted for ≈2 days and peaked at 48 h (**Figure** **3**a). The rapid up‐regulation of miR162 was consistent with the results of small RNA sequencing analysis (Figure S3a, Supporting Information). In contrast, no significant changes in miR162 levels were observed in young stems grown under upland conditions (Figure S3a, Supporting Information). In old stems, the increase in miR162 levels occurred later, at 24–48 h after water submergence, but not as rapidly as in young stems (Figure 3a). Similarly, no up‐regulation of miR162 was seen in old stems grown under upland conditions (Figure S3a, Supporting Information). This rapid responsiveness of miR162 to water submergence suggests that its up‐regulation might be involved in the stem elongation of *A. philoxeroides* in aquatic environments.

To determine whether the accumulation of miR162 contributes to its aquatic habitat adaptation in *A. philoxeroides*, we attempted to knock down miR162 levels using the TRV‐based VIGS system. In *A. philoxeroides*, two genes encode *MIR162*: *MIR162a* and *MIR162b*. To investigate whether *AphMIR162a*, *AphMIR162b*, or both respond to water submergence by inducing the production of mature miR162, we conducted RT‐qPCR analyses to examine the levels of precursor miR162 (pri‐miRNA). The results showed that the expression of pri‐miR162b, but not pri‐miR162a, was upregulated under water submergence (Figure 3b). Subsequently, we performed knockdown experiments targeting miR162 using the TRV‐based VIGS system. Two fragments, each specific to either *AphMIR162a* or *AphMIR162b*, were separately cloned into the pTRV2 vector. These plasmids were introduced into the leaves of *A. philoxeroides*, and the efficiency of miR162 knockdown was assessed by measuring mature miR162 levels through RT‐qPCR analyses. As shown in Figure 3c, the levels of pri‐miRNA were significantly reduced in samples inoculated by either the pTRV2‐AphMIR162a or pTRV2‐AphMIR162b constructs compared to the mock treatment. This reduction indicates that the introduction of these constructs effectively suppressed pri‐miRNA levels. Correspondingly, the levels of mature miR162 were also markedly downregulated following the introduction of the pTRV2‐AphMIR162a or pTRV2‐AphMIR162b plasmid into the leaves of *A. philoxeroides* (Figure 3c). These findings are in line with the observed strong responsiveness of *MIR162b* transcription to water submergence, as illustrated in Figure 3b. We then compared the overall growth phenotypes of *A. philoxeroides* plants inoculated with the mock control, pTRV2‐AphMIR162a, or pTRV2‐AphMIR162b. The results show that the plants inoculated with the pTRV2‐AphMIR162b plasmid exhibited a significantly reduced height compared to the mock control (Figure 3d). In contrast, the plants expressing pTRV2‐AphMIR162a showed only a slight reduction in height (Figure 3d). Statistical analyses of stem length confirmed that the introduction of pTRV2‐AphMIR162b significantly inhibited the elongation of internode 1 and had a slight inhibitory effect on the elongation of internodes 2 and 3 (Figure 3e). In contrast, the effects of knocking down *AphMIR162a* were very slight (Figure 3e). This suggests that the suppression of *AphMIR162b* may have a more pronounced impact on plant growth compared to the relatively minor effect of *AphMIR162a*, highlighting potential differences in their roles in regulating plant development and stress responses. To determine whether the effects of miR162 on stem elongation are due to changes in cell expansion, we measured the lengths of surface cells on the stems. As shown in Figure S3b,c (Supporting Information), the cell lengths in the pTRV2‐AphMIR162b group were noticeably shorter than those in the mock control.

To investigate how miR162 promotes stem elongation under flooding stress, we focused on light, hormone, and cell elongation‐related genes previously reported to be upregulated by waterlogging treatment.^[^ ^15^, ^16^ ^]^ We then used RT‐qPCR to examine the expression of these genes following miR162 knockdown. As shown in Figure S3d (Supporting Information), positive regulators such as *PIF7, BZR1, GID1B*, and several expansin genes (*EXPA1, EXPA3, EXPA4*, and *EXPA5*) were significantly downregulated, while negative regulators like GAI and RGL1 were substantially upregulated. These coordinated changes in gene expression, spanning multiple hormonal pathways and cell wall modification components, offer mechanistic insights into the suppression of stem elongation following miR162 knockdown, underscoring miR162's pivotal role in integrating various signaling pathways during the submergence response. Collectively, these results demonstrate that miR162 plays a critical role in promoting stem elongation, which is essential for the adaptation of *A. philoxeroides* to aquatic habitats.

### 2.4 Responsiveness of miR162 to Aquatic Habitats is Specific to Invasive Plant *A. philoxeroides* Rather than Noninvasive Plant *A.pungens*

**Figure 4:**
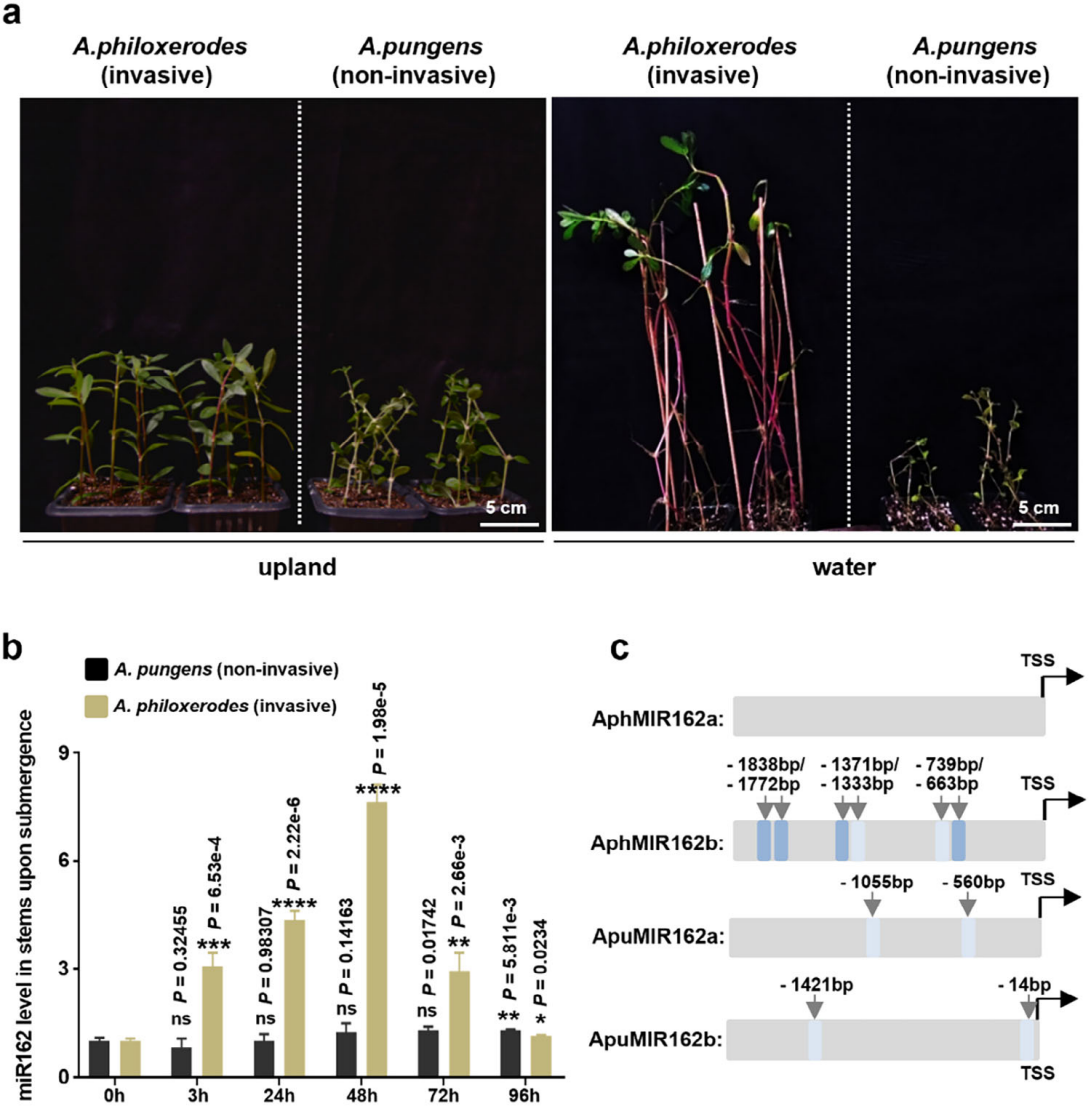
Upregulation of *AphMIR162b* is associated with invasion capacity in *A. philoxeroides*. a) Plant height comparison of upland (two left panels) and water‐logged (two right panels) *A. philoxeroides* with *A. pungens* for 20 days after submergence treatment. b) miR162 levels at different time points in stems of *A. philoxeroides* and *A. pungens* after submergence treatment. Data are presented as mean ± standard errors (SE) from three biological replicates. ****p < 0.0001, ***p < 0.001, **p < 0.01, and *p < 0.05 were obtained using Student's *t*‐test. ns, not significant. c) A schematic diagram shows the distribution of AREs (Anaerobic Responsive Elements) in the promoter sequences of MIR162 genes in invasive species *A. philoxeroides* and noninvasive species *A. pungens*. The dark blue shading highlights the conserved ARE sequence (VVAAACCAVV), where “V” represents A, C, or G. The light blue shading represents the degenerate ARE sequence (AAACCA), where possible T can be the flanking nucleotides. The arrows indicate the locations of the AREs upstream of the TSS (transcription start sites).

As previously mentioned, *A. pungens* is evolutionarily closely related to *A. philoxeroides*, with both species native to South America. However, upon their introduction to East Asia in the 1930s, *A. philoxeroides* rapidly established itself as one of China's top 100 invasive plants due to its robust asexual propagation and its adaptability to various habitats. In contrast, *A. pungens* maintained its preference for upland conditions in Asia. As shown in **Figure** **4**a, both species grow similarly in upland environments. However, when submerged, the stems of *A. philoxeroides* typically elongate and break the water's surface within 1–2 days, whereas *A. pungens* lacks this capability and ultimately withers and dies in such conditions (Figure 4a). To investigate whether the induction of miR162 contributes to the differences in invasive potential between *A. philoxeroides* and *A. pungens*, we conducted RT‐qPCR analyses to measure miR162 levels at various time points before and after water submergence. As expected, *A. philoxeroides* showed a significant increase in miR162 levels at 3 h after submergence, peaking at 48 h (Figure 4b), which aligns with previous observations (Figure 3a). In contrast, miR162 levels in *A. pungens* remained consistently low and stable, even 72 h after submergence (Figure 4b). This indicates that miR162 is not responsive to aquatic habitats in *A. pungens*.

To explore why miR162 is induced by water submergence in *A. philoxeroides* but not in *A. pungens*, we analyzed the promoter sequences (≈2 kb upstream of the transcription site, TSS) of *A* *ph* *MIR162a* and *A* *ph* *MIR162b* in both species for specific cis‐acting regulatory elements using the PlantCARE database.^[^ ^17^ ^]^ These analyses identified that the promoter region of *AphMIR162b* in *A. philoxeroides* contains four highly conserved Anaerobic Responsive Elements (AREs, VVAAACCAVV, where V represents A, C, or G) (Figure 4c; Figure S4, Supporting Information, highlighted in dark blue shadow) and two degenerate AREs (AAACCA, with possible T as the flanking nucleotides) (Figure 4c; Figure S4, Supporting Information, highlighted in light blue shadow). In contrast, the promoter regions of *A* *ph* *MIR162a* in *A. philoxeroides* and both *AphMIR162* genes in *A. pungens* were found to either lack ARE entirely or contain only 1 to 2 degenerate ARE elements (Figure 4c; Figure S4, Supporting Information). AREs are recognized for their roles in mediating responses to anaerobic conditions.^[^ ^18^, ^19^, ^20^, ^21^ ^]^ The presence of AREs suggests that *A* *ph* *MIR162b* may be specifically regulated by low‐oxygen environments, potentially contributing to the submergence survival strategies. Given that anaerobic environments are a hallmark of aquatic habitats,^[^ ^22^ ^]^ the presence of more conserved AREs in the promoter of *A* *ph* *MIR162* *b* likely plays a critical role in the adaptation of *A. philoxeroides* to such environments.

### 2.5 Nanoparticle‐Delivered Antisense RNA Oligonucleotides of miR162 Inhibits Submergence‐Induced Stem Elongation of *A. philoxeroides*

**Figure 5:**
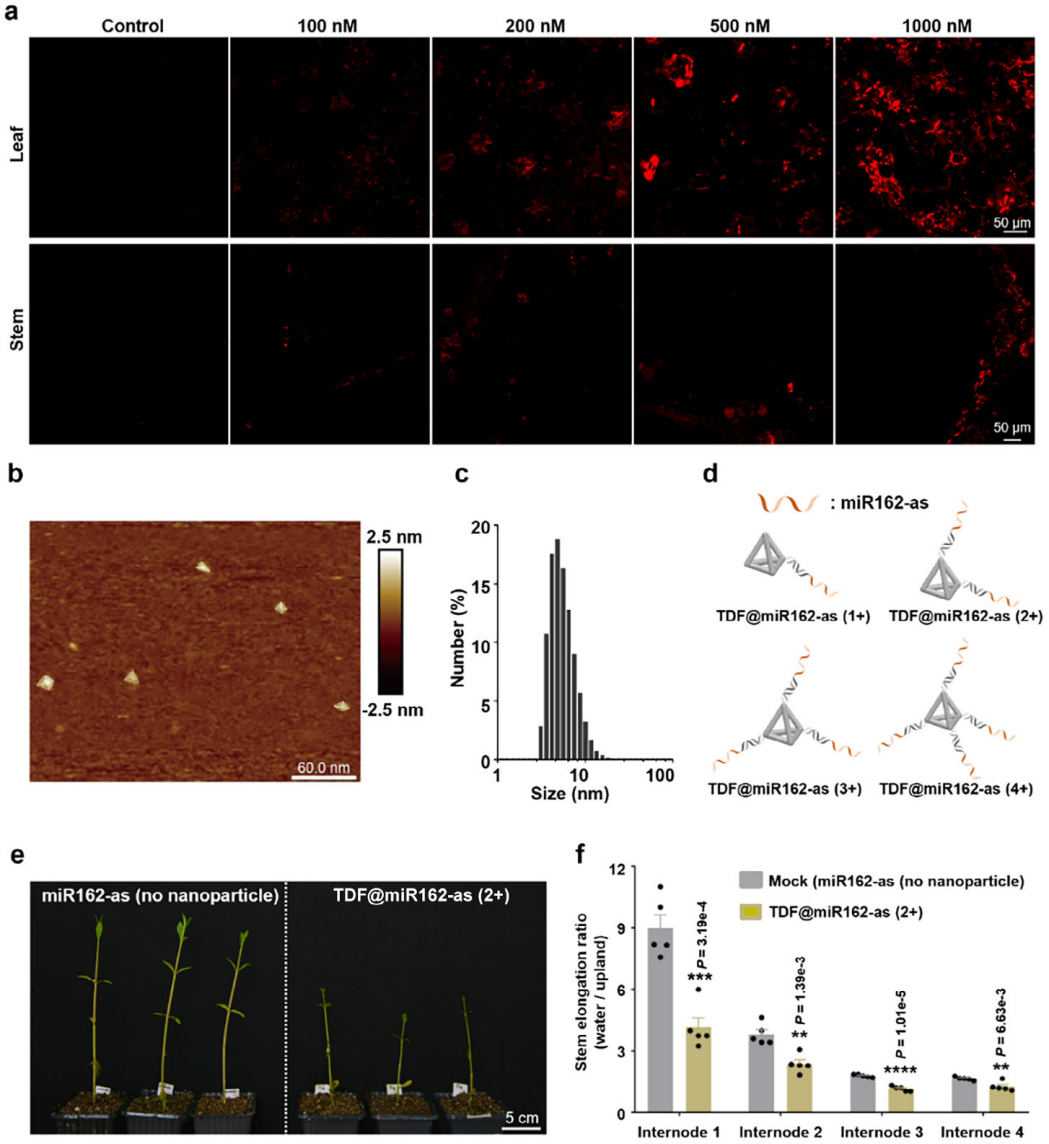
Nanoparticle‐mediated delivery of antisense oligonucleotides of miR162 inhibits submergence‐induced stem elongation in *A. philoxeroides*. a) The localization and transportation of Cy3‐labelled TDF within *A. philoxeroides*. Scale bars = 50 µm. b) AFM characterization image of TDF. Scale bars = 60 nm. c) DLS analysis of TDF. d) Illustration of the design and assembly of one, two, three, and four miR162‐as onto TDF structures to generate TDF@miR162‐as (1+), TDF@miR162‐as (2+), TDF@miR162‐as (3+) and TDF@miR162‐as (4+). e) Plant height comparison of water‐logged *A. philoxeroides* after delivery by TDF nanoparticle‐packaged as‐miR162 (TDF@miR162 (2+)) and the mock control (miR162‐as with no nanoparticle). Scale bars = 5 cm. f) Statistical analyses of the elongation ratio of internode in (e). Five plants were analyzed for each group, and each dot represents one internode. ****p < 0.0001, ***p < 0.001 and **p < 0.01 were obtained by Student's *t*‐test.

Given that the up‐regulation of miR162 is essential for stem elongation in *A. philoxeroides* during water submergence, it allows the plant to grow above the water surface and contributes to its survival in aquatic habitats. Thus, there is potential to develop a biocontrol method based on the sequestration of miR162 activity. To pursue this objective, we aimed to establish an innovative biocontrol strategy utilizing RNA‐based pesticides to manage the invasion of *A. philoxeroides*. This approach could effectively disrupt the growth of this invasive species, offering a targeted solution for controlling its spread in aquatic environments. Previous research has demonstrated that nanoparticle‐packaged RNA oligonucleotides exhibit excellent programmability, high delivery efficiency, and their slow‐release characteristics ensure the stability of the delivered RNA.^[^ ^23^, ^24^, ^25^, ^26^ ^]^ Building upon these findings, we investigated the application of nanoparticle‐packaged RNA oligonucleotides in *A. philoxeroides*. To track the localization of these nanoparticles within *A. philoxeroides*, we used Cy3‐labeled tetrahedral DNA framework (TDF) to trace its transport. By injecting the nanoparticles into the leaves and observing the distribution of Cy3 fluorescence 12 h post‐injection, we confirmed that TDF could penetrate leaf cells (**Figure** **5**a). Additionally, we detected Cy3 fluorescence in the stem adjacent to the injected leaves at 48 h post‐injection, indicating that TDF was transported downward from the leaves to the stem, fulfilling its intended delivery function (Figure 5a). To assess the transport efficiency of TDF in *A. philoxeroides*, we established different concentration gradients. The results showed that higher concentrations (from 100 to 1000 nM) led to greater amounts of TDF being delivered and transported into the plants (Figure 5a). Based on a previous finding^[^ ^27^ ^]^ and considering both effectiveness and cost, a final concentration of 200 nM miR162‐as was chosen as sufficient for further delivery and growth inhibition studies. We further utilized atomic force microscopy (AFM) and dynamic light scattering (DLS) to analyze their size and morphology (Figure 5b,c), revealing several 3D tetra‐structures with an average size of ≈5 nm. Thus, these results indicate that the method of delivering RNA using TDF nanoparticles is effective in *A. philoxeroides*.

To further validate this approach and investigate its biological applications, we conducted experiments to assess the effects of TDF‐delivered antisense miR162 oligonucleotides on stem elongation in *A. philoxeroides* in aquatic habitats. As illustrated in Figure 5d, we designed and synthesized four types of TDF structures, each loaded with one, two, three, or four antisense RNA oligonucleotides that are complementary to miR162 (miR162‐as) that were accommodated at the vertices of the TDF. We named these structures TDF@miR162‐as (1+), TDF@miR162‐as (2+), TDF@miR162‐as (3+), and TDF@miR162‐as (4+), respectively. In detail, the miR162‐as oligonucleotides were designed with a poly(A) tail, allowing them to hybridize with the poly(T) sticky ends extended from the vertices of the TDF. We characterized the successful assembly of the TDF nanostructure and the precise loading of miR162‐as using gel electrophoresis. The bands gradually shifted upward as more miR162‐as oligonucleotides hybridized at the vertices (Figure S5a, Supporting Information), indicating successful loading of the miR162‐as. Subsequently, we inoculated the leaves of *A. philoxeroides* with these TDF‐loaded antisense RNA oligonucleotides targeting miR162 and normalized the miR162‐as with a final concentration of 200 nM. After 10 days of water submergence, we observed the phenotypes related to stem elongation. The results indicated that all TDFs carrying antisense miR162 oligonucleotides exhibited a significant inhibitory effect on stem elongation compared to the two negative controls: naked antisense miR162 oligonucleotides and phosphate‐buffered saline (PBS) alone (Figure 5e; Figure S5b, Supporting Information). Notably, we found that the TDFs assembled with two antisense miR162 oligonucleotides exhibited the strongest inhibitory effects on stem elongation (Figure S5b, Supporting Information). We attribute this effect to the conformation of the miR162‐as on the TDF, as well as the increased steric hindrance encountered by miR162 when recognizing TDF@miR162‐as (3+) and TDF@miR162‐as (4+). These findings suggest that the utilization of nanoparticle‐delivered antisense miR162 oligonucleotides is a promising strategy for controlling the growth of *A. philoxeroides* in aquatic environments.

To further validate the impact of miR162 knockdown, we employed target‐directed miRNA degradation (TDMD)^[^ ^28^, ^29^ ^]^ to investigate the effects of nanoparticle‐delivered antisense miR162 on endogenous miR162 levels in *A. philoxeroides* under flooding. We used TDF to deliver an antisense sequence of miR162 with an additional three‐nucleotide insertion (GUA) at positions 10–11, which corresponds to the well‐known seed sequences of miRNA. This modification created a simulated target, facilitating the degradation of miR162 through the TDMD mechanism. We then quantified miR162 levels using stem‐loop RT‐qPCR and observed a more significant reduction in miR162 expression compared to the control. Concurrently, stem elongation in the plants was markedly inhibited (Figure S5c–e, Supporting Information). These results demonstrate that TDF@miR162‐asTDMD (2+) effectively knocks down miR162, thereby suppressing stem elongation in *A. philoxeroides* under flooding stress. Collectively, we conclude that inhibition of either miR162 biogenesis or activity effectively prevents stems from protruding above the water surface. This highlights the potential for targeting miR162 as a strategic approach to control the growth and spread of this invasive species in aquatic habitats.

## 3 Discussion

miRNAs are pivotal regulatory molecules that play crucial roles in various biological processes, including development and the response to environmental stress.^[^ ^6^, ^30^ ^]^ In the context of *A. philoxeroides*, the phenotypic flexibility observed in invasive aquatic habitats is not attributed to changes in genetic information. Instead, epigenetic regulation emerges as a key mechanism underpinning this adaptability. In this study, we identified a specific microRNA, miR162, which plays a crucial role in the establishment of its invasive capacity in aquatic environments. We present three lines of evidence to suggest that inhibiting miR162 could be a feasible strategy for the biocontrol of *A. philoxeroides*. First, we demonstrate that a deficiency in the entire miRNA pathway, including both miRNA biogenesis machinery and miRNA activity, can effectively impede the growth of *A. philoxeroides* in aquatic habitats. Second, we show that the knockdown of miR162 levels using the TRV‐based VIGS silencing system is sufficient to inhibit the growth of *A. philoxeroides* in these environments. Lastly, we successfully applied nanoparticle‐delivered antisense miR162‐based pesticides to disrupt the growth of *A. philoxeroides* in aquatic habitats. In the future, we believe that a combination of approaches will often be more effective in controlling this invasive species. For example, after a round of mechanical cutting to reduce the bulk of the *A. philoxeroides* population, miRNA‐based treatments could be applied to target the remaining plants at the molecular level. This approach would reduce competition for resources, minimize the amount of miRNA‐based agents needed, and address the underlying genetic processes that drive regrowth. Additionally, combining miRNA regulation with traditional strategies can help mitigate some of the negative impacts associated with any single method. For example, using a low dose of herbicides in combination with miRNA‐mediated gene silencing could reduce the environmental footprint of chemical control while still achieving effective weed management.

In our experiments, the use of TDF nanoparticles exhibits the ability to penetrate plant cells directly, which enhances the stability of the antisense miR162 that they carry. As a result, TDF nanoparticles demonstrated a significantly stronger efficacy in sequestering miR162. Notably, when two antisense miR162 molecules were attached to the TDF nanoparticles, the efficiency was higher than that observed with four antisense miR162 molecules. This phenomenon may be explained by the saturation of the binding sites on the tetrahedral nanoparticles, which could hinder further entry into plant cells when overloaded. Overall, these findings indicate that the selection of delivery systems and the optimization of RNA load are critical for the effective application of antisense miRNAs in controlling the growth of *A. philoxeroides* and potentially other invasive species. Further exploration of nanoparticle design and its interaction with plant cells could lead to more effective biocontrol strategies to manage invasive species. Given that foliar spraying is the most practical and efficient method for RNA pesticide application, combining it with other approaches, such as mechanical cutting, will be considered for enhanced effectiveness.

Nanoparticle‐delivered antisense RNA oligonucleotides targeting miR162 effectively inhibit stem elongation in *A. philoxeroides* when exposed to aquatic habitats. This intervention suggests that the suppression of miR162 disrupts the plant's normal growth responses to submerged conditions, potentially affecting its invasive potential and adaptability in aquatic environments. This targeted approach highlights the role of miR162 in regulating stem elongation and could offer insights into managing the growth of *A. philoxeroides* in its invasive habitats. Given that *A. philoxeroides* has become an invasive species across Asia, North America, and Australia due to its high reproductive potential and adaptability, we believe that nanoparticle‐delivered miR162 antisense oligonucleotide pesticides offer a viable strategy for its global control. Additionally, our findings may provide clues for generating crops via molecular breeding that can tolerate flooding resilience.

## 4 Conclusion

The rapid spread of invasive species poses a major challenge to biodiversity conservation. *Alternanthera philoxeroides*, native to South America, has globally infiltrated various habitats due to its strong adaptability, emerging as a significant ecological threat. *A. philoxeroides* exhibits several remarkable adaptive mechanisms that contribute to its success as an invasive species. One key adaptation is its high tolerance to aquatic habitats, allowing it to rapidly grow out of the water by significantly increasing its stem internode length within a few days. Although mechanical clearance and chemical pesticide spraying have shown significant improvements in controlling *Alternanthera philoxeroide*s, it is essential to note that these methods come with considerable annual costs and environmental concerns related to chemical contamination. Therefore, understanding the mechanism by which *A. philoxeroides* can rapidly protrude its stem out of the water's surface to survive submergence is crucial, and the development of an effective biological control strategy for controlling this invasive species is urgently needed. In this study, we identified miR162 plays a vital role in *A. philoxeroides*' ability to thrive in aquatic environments and establish its invasive capacity. We provide two lines of evidence to suggest that inhibiting miR162 could be a promising approach for controlling the spread of *A. philoxeroides* through biological means. First, we show that knockdown of miR162 levels using the TRV‐based VIGS silencing system is sufficient to inhibit the stem elongation of *A. philoxeroides* in these environments. Second, we successfully applied nanoparticle‐delivered antisense oligonucleotides of miR162 to disrupt the stem elongation of *A. philoxeroides* in aquatic habitats. In summary, this study shows that inhibiting miR162 can effectively control the invasive capacity of *A. philoxeroides* in aquatic environments, and suggests that these findings may have implications for the development of novel biocontrol strategies against this invasive species.

## 5 Experimental Section

### Plant Materials

The invasive population of *A. philoxeroides* used in this study was collected in Zhuji, Zhejiang Province, China (120°20′ E, 29°40′ N). The collected materials were grown in nutrient‐rich soil and maintained in a greenhouse at Fudan University, Shanghai (121°29′ E, 31°14′ N). Stem fragments from a single individual plant were cut from ramets with similar diameters and planted in plastic plates (dimensions: upper diameter 16 cm, lower diameter 12.5 cm, height 13 cm), which were filled with 1.5 L of a 1: 1 soil mixture (black soil: sand). Following the appearance of the first two new leaves, plants of similar size were individually transplanted into sand pots and grown under common garden conditions in the greenhouse for a duration of 2 months prior to the initiation of the two experimental treatments. For the submergence treatment, the plants were fully immersed in 50 cm‐deep water, while the terrestrial control group received 1 L of water daily to maintain wet but well‐drained soil conditions. *A. pungens* were collected from Yunnan, China. The seeds were pre‐germinated for 3 days before the seedlings were individually transplanted into sand pots and maintained under the same conditions as *A. philoxeroides*. For *Arabidopsis thaliana* plants, seeds of the wild type Columbia‐0 (Col‐0), as well as various mutants including *hyl1‐2* (SALK_064863),^[^ ^31^ ^]^ *ago1‐27*,^[^ ^32^ ^]^ *mir159abc* (SAIL_430_F11 for *mir159a*; SAIL_770_GO5 for *mir159b*; and SAIL_248_G11 for *mir159c*),^[^ ^33^ ^]^ *mir164abc* (Cs65828),^[^ ^34^ ^]^ and *myb33 myb65* (CS851168/*myb33*; crispr‐cas9/*myb65*)^[^ ^7^ ^]^ were germinated on 1/2 Murashige and Skoog (MS) medium and grown at 22 °C under a 16‐h light/8‐h dark photoperiod. After 4 days on solid 1/2 MS medium, seedlings were transferred to liquid MS medium, ensuring that the seedlings remained completely submerged. They were allowed to grow for an additional ten days, after which phenotypic observations were conducted.

### Plasmid Construction

The vectors pTRV2 and pTRV2‐GFP,^[^ ^11^ ^]^ provided by Prof. Feng Li from Huazhong Agricultural University in China, were utilized for the construction of the pTRV2‐AphPDS, pTRV2‐AphDCL1, pTRV2‐AphAGO1, pTRV2‐AphMIR162a and pTRV2‐AphMIR162b vectors. DNA sequences were amplified from *A. philoxeroides* and subsequently cloned into the pTRV2 vector. Details of the primers used for amplification can be found in Table S2 (Supporting Information).

### Inoculation of TRV‐Based VIGS into *A. philoxeroides*

Overnight cultures of Agrobacterium expressing the TRV2 vectors and the constitutive vector of TRV1^[^ ^12^ ^]^ were harvested by spinning, respectively, and the pellets were re‐suspended in a solution containing 10 mM MES (2‐(N‐morpholino) ethanesulfonic acid), 10 mm MgCl₂, and 200 mM acetosyringone (AS) to a final optical density (OD_600_) of 1.5. Then, the TRV2 vector‐containing solution was mixed with the TRV1 plasmid‐containing in a 1:1 ratio with volumes. This agrobacterium suspension was then infiltrated into each leaf of *A. philoxeroides*, with at least 10 plants inoculated for each construct.

### RNA Isolation, Library Preparation, and Sequencing

Stem samples were collected from young (1‐2 internodes) and old (3‐4 internodes) plants at multiple time points: 0, 3, 24, 48, 72, and 96 h, both in terrestrial conditions and following waterlogging treatment. Each treatment and time point consisted of three biological replicates, with each replicate including three individual plants. Total RNA was extracted from the stems using TRIzol reagent (Invitrogen, USA). The RNA concentration was determined using a Qubit fluorometer (Thermo, USA), while the RNA quality was assessed using a Qsep100 system with an R1 RNA Cartridge Kit (BiOptic Inc., China). For small RNA sequencing, 1 µg of total RNA from each sample was used for library preparation, employing the VAHTS Small RNA Library Prep Kit for Illumina V2 (Vazyme, NR811‐01) according to the manufacturer's instructions. The purified small RNA libraries were sequenced using the Illumina NovaSeq 6000 platform.

### Analyses of Small RNA Sequencing Data

The sRNA‐Seq libraries were analyzed using computational methods reported in previous studies.^[^ ^35^, ^36^ ^]^ Initially, the 3′ adapters from all sequenced reads were clipped to obtain the resulting small RNA reads. Reads shorter than 18 nucleotides were discarded, and redundant reads were removed to generate a unique set of reads with associated counts. To identify homologs of conserved miRNAs in *Alternanthera philoxeroides*, mature miRNA sequences from other plant species were downloaded from miRBase (version 22),^[^ ^37^ ^]^ and unique mature miRNA sequences were obtained. These unique miRNAs were subjected to alignment against the *A. philoxeroides* genome using NCBI BLASTN (version 2.2.26),^[^ ^38^ ^]^ with alignment parameters set to “‐m 8 ‐e 0.01.” For every matched locus, flanking regions of 80, 130, and 180 nt both downstream and upstream were extracted, and secondary structures for these sequences were predicted using RNAfold.^[^ ^39^ ^]^ Based on the predicted fold‐back structures and reported family members from other plants, the existence of mature miRNAs located on the same arms of the hairpins was examined. To validate these precursor sequences, MIRcheck was utilized to ensure that the sequences met specific criteria: a maximum of 2 bulged nucleotides, fewer than or equal to five mismatches or asymmetrically unpaired nucleotides, and no more than three continuous mismatches within the mature miRNA. Subsequently, small RNA sequencing reads were aligned to the pre‐miRNA candidates to observe the distribution of small RNA reads among these candidates, employing the criteria outlined in a previous finding.^[^ ^40^ ^]^ The frequencies of mature miRNAs in the different small RNA sequencing profiles were calculated by aligning the reads to the mature miRNAs using NCBI BLASTN (version 2.2.26)^[^ ^37^ ^]^ with parameters “‐S 1 ‐m 8 ‐e 0.01,” and results were normalized to Reads Per Ten Million (RPTM) sequencing tags. To compare expression levels of miRNAs among the different sample groups, the edgeR software package was utilized.^[^ ^41^ ^]^ Specifically, comparisons were made using young and old stem samples from both terrestrial and waterlogged conditions at time points of 3, 24, 48, 72, and 96 h against the control samples collected at 0 h. miRNA was considered significantly deregulated if the corrected *p*‐value was less than 0.05.

### miRNA Detection By Stem‐Loop RT‐qPCR

A quantity of 0.5 µg of DNase I‐treated RNA was subjected to first‐strand cDNA synthesis in 20 µL reaction with RT buffer (50 mmol L−1 Tris‐Cl pH 8.3, 75 mmol L−1 KCl, 3 mmol L−1 MgCl_2_, 2 U µL−1 RNase inhibitor), 1 µL dNTPs (10 mM), SuperScript IV reverse transcriptase and 1µM each stem‐loop primers designed for the miRNAs of interest and the U6. The reaction mixture was incubated at 16 °C for 30 min (this step allows the stem‐loop primer to anneal), followed by pulsed RT of 60 cycles at 30 °C for 30 s, 42 °C for 30 s, and 50 °C for 1 s, the reverse transcriptase was inactivated by heating at 85 °C for 5 min as described previously.^[^ ^42^, ^43^ ^]^ The resulting cDNA was diluted with RNase‐free water to the concentration suitable for qPCR, typically 1:5 to 1:10.

### Quantitative Real‐Time PCR

Total RNA was extracted using TRIzol. The PrimeScript II Reverse Transcriptase (Takara, 2690B) was used to reverse‐transcribe RNA into cDNA following the manufacturer's protocol, then the cDNAs were subjected to real‐time qPCR using the CFX Connect Real‐Time System (Bio‐Rad, USA). Each reaction was replicated three times. The relative expression levels were calculated using the 2^−ΔΔCt^ method. The primers are listed in Table S2 (Supporting Information).

### Preparation of Tetrahedral DNA Framework (TDF) Nanoparticle

The tetrahedral DNA framework (TDF) with an edge length of 13 bp was synthesized by mixing four designed strands ((T13‐a, b, c, d, see detailed sequences in Table S2, Supporting Information.) synthesized by Sangon Biotech in equimolar ratios in TM buffer (10 mM Tris‐HCl, 5 mM MgCl_2_, pH 8.0), following the previously reported methods.^[^ ^23^, ^24^ ^]^ The solution was heated to 95 °C for 10 min and then quickly cooled to 4 °C for 30 min. For TDF aimed at loading 1, 2, 3, or 4 sticky ends for miR162‐as, T13‐a, b, c, and d were replaced with 10T‐T13‐a, b, c, and d.

### Assembly of miR162‐as onto TDFs

First, the experimental steps outlined above to synthesize TDFs with suspended polyT sticky ends were followed. PolyA‐miR162‐as was then added to the TDFs in a calculated molar ratio and annealed using the same procedure. Subsequently, PolyA‐miR162‐as with polyA tails were site‐specifically coupled to TDFs through hybridization with free polyT sequences displayed at specific vertex sites. This approach allowed to generation of TDFs containing one, two, three, or four miR162‐as.

### Infiltration of Leaves with TDF‐Loaded miR162‐as

In brief, the process involves the following steps: using a sterile needle to make a short incision on the abaxial surface of the *A. philoxeroides* leaf lamina. It is crucial that the incision is shorter than the tip of a syringe to minimize damage to the plant tissue. Then, using a 1‐mL needleless syringe, gently infiltrate 100 µL of the prepared nanostructure solutions into the incision site. After the injection, immediately submerge the treated plants in water for ten days. Following this period, compare the elongation of the stems between treated and control plants. Measure the stem lengths to assess the impact of the nanostructure treatments on plant growth.

### Native Polyacrylamide Gel Electrophoresis

The successful assembly of TDF and TDF loaded with miR162‐as were characterized using 10% native polyacrylamide gel electrophoresis (PAGE) with 1×TAE buffer running at 85 V for ≈80 min in the electrophoresis apparatus instrument. Then, the gels were stained with 1 × GelRed nucleic acid dye for ≈2 min and scanned under UV light. Image analysis was performed with the software Image J.

### Atomic Force Microscopy

For AFM images of TDFs, 10 µL of 25 nm TDFs were dropped onto the mica surface for 10 min. 20 µL 1×TM buffer was dropped onto the mica surface. The image of TDFs was obtained by Bruker multimode 8.

### Dynamic Light Scattering Analysis

Three hundred microliter TDF solution (1 µM) was added into a plastic cuvette, then the hydrodynamic diameters were measured by a Malvern Zetasizer Nano‐ZS instrument (Malvern Instruments, UK) equipped with a He–Ne laser (633 nm).

### Microscopy

GFP fluorescence microscopy analyses were carried out with an Olympus FV3000 laser confocal microscope, excitation and emission wavelengths were 488 nm and 500 to 550 nm respectively. Cy3 fluorescence signals (emission range from 570 to 620 nm) were collected by Lecia stellaris 5 under the 561 nm excitation wavelength. The captured images were processed using Adobe Photoshop and Image J software.

### Scanning Electron Microscope (SEM)

Stems collected from relative *A. philoxeroides* were carefully placed on conductive tape for analysis using a Hitachi TM3000 scanning electron microscope. The parameters were set as follows: Accelerating Voltage = 15 000 Volt, Magnification = 200, Brightness = 2243, Contrast = 4095. The captured pictures were used to measure the lengths of stem epidermal cells through Image J software.

### Predicting the Cis‐Regulatory Elements Located in Promoter Regions

The PlantCARE (https://www.bioinformatics.psb.ugent.be/webtools/plantcare/html/) online database was used to predict cis‐regulatory elements located in the promoter sequences of *MIR162* genes in *A. philoxeroides* and *A. pungens*.

### Quantification and Statistical Analysis

Statistical methods for assessing peak overlap included one‐sided permutation overlap tests or two‐sided Fisher's exact tests, respectively. All boxplots, bar plots, and line charts were generated by R or GraphPad Prism 9.5. Statistical significance was determined by one‐way ANOVA or Student's *t*‐test.

## Conflict of Interest

The authors declare no conflict of interest.

## Author Contributions

Q.H. and E.Q. contributed equally to this work and co‐first authors. B.Z. and J.Y. conceptualized the study. Q.H. performed most experiments. E.Q. and H. Z. finished the preparation of nanoparticles. X. L. and Y.Z. finished miRNome analysis. R.Q. and Q.T. helped with sample preparation for small RNA seq analysis. B.Z. wrote the manuscript, and Q.H., E.Q., Y.Z., H. Z, and J.Y. revised the manuscript.

## Supporting information



Supporting Information

Supplemental Table 1

Supplemental Table 2

## Data Availability

The data that support the findings of this study are openly available in NCBI GEO at https://www.ncbi.nlm.nih.gov/geo/query/acc.cgi?acc=GSE283087, reference number 283087.

## References

[1] Y. Chen , Y. Zhou , T. F. Yin , C. X. Liu , F. L. Luo , PLoS One 2013, 8, 81456. 10.1371/journal.pone.0081456 PMC3841148 24303048

[2] N. Wang , F. H. Yu , P. X. Li , W. M. He , F. H. Liu , J. M. Liu , M. Dong , Ann. Bot. 2008, 101, 671. 18250108 10.1093/aob/mcn005 PMC2710179

[3] Y. Geng , R. D. van Klinken , A. Sosa , B. Li , J. Chen , C. Y. Xu , Front. Plant. Sci. 2016, 7, 213. 26941769 10.3389/fpls.2016.00213 PMC4764702

[4] L. Gao , Y. Geng , B. Li , J. Chen , J. Yang , Plant Cell Environ. 2010, 33, 1820. 20545886 10.1111/j.1365-3040.2010.02186.x

[5] G. Li , Y. Deng , Y. Geng , C. Zhou , Y. Wang , W. Zhang , Z. Song , L. Gao , J. Yang , Front. Plant Sci. 2017, 8, 2078. 29259617 10.3389/fpls.2017.02078 PMC5723390

[6] K. Rogers , X. Chen , Plant Cell 2013, 25, 2383. 23881412 10.1105/tpc.113.113159 PMC3753372

[7] H. Yang , Y. Zhao , Z. Lin , T. Jiang , Q. Hu , G. Ren , B. Zheng , Seed Biol. 2024, 3, 017.

[8] C. Bai , P. Wang , Q. Fan , W. D. Fu , L. Wang , Z. N. Zhang , Z. Song , G. L. Zhang , J. H. Wu , Front. Plant Sci. 2017, 8, 1579. 28955366 10.3389/fpls.2017.01579 PMC5601067

[9] F. Ratcliff , A. M. Martin‐Hernandez , D. C. Baulcombe , Plant J. 2001, 25, 237. 11169199 10.1046/j.0960-7412.2000.00942.x

[10] G. Shi , M. Hao , B. Tian , G. Cao , F. Wei , Z. Xie , Front. Plant Sci. 2021, 12, 671091. 34149770 10.3389/fpls.2021.671091 PMC8212136

[11] Y. Zhou , Y. Deng , D. Liu , H. Wang , X. Zhang , T. Liu , J. Wang , Y. Li , L. Ou , F. Liu , X. Zou , B. Ouyang , F. Li , Plant Biotechnol. J. 2021, 19, 2398. 34628716 10.1111/pbi.13724 PMC8633498

[12] Y. Liu , M. Schiff , R. Marathe , S. P. Dinesh‐Kumar , Plant J. 2002, 30, 415. 12028572 10.1046/j.1365-313x.2002.01297.x

[13] M. Senthil‐Kumar , K. S. Mysore , Nat. Protoc. 2014, 9, 1549. 24901739 10.1038/nprot.2014.092

[14] D. Jiang , C. Yin , A. Yu , X. Zhou , W. Liang , Z. Yuan , Y. Xu , Q. Yu , T. Wen , D. Zhang , Cell Res. 2006, 16, 507. 16699546 10.1038/sj.cr.7310062

[15] L. Gao , Y. Geng , H. Yang , Y. Hu , J. Yang , Front Plant Sci. 2015, 6, 991. 26617628 10.3389/fpls.2015.00991 PMC4641913

[16] A. Minami , K. Yano , R. Gamuyao , K. Nagai , T. Kuroha , M. Ayano , M. Nakamori , M. Koike , Y. Kondo , Y. Niimi , K. Kuwata , T. Suzuki , T. Higashiyama , Y. Takebayashi , M. Kojima , H. Sakakibara , A. Toyoda , A. Fujiyama , N. Kurata , M. Ashikari , S. Reuscher , Plant Physiol. 2018, 176, 3081. 29475897 10.1104/pp.17.00858 PMC5884608

[17] M. Lescot , P. Dehais , G. Thijs , K. Marchal , Y. Moreau , Y. Van de Peer , P. Rouze , S. Rombauts , Nucleic Acids Res. 2002, 30, 325. 11752327 10.1093/nar/30.1.325 PMC99092

[18] J. C. Walker , E. A. Howard , E. S. Dennis , W. J. Peacock , Proc. Natl. Acad. Sci. USA 1987, 84, 6624. 16578816 10.1073/pnas.84.19.6624 PMC299135

[19] M. R. Olive , J. C. Walker , K. Singh , E. S. Dennis , W. J. Peacock , Plant Mol. Biol. 1990, 15, 593. 2102377 10.1007/BF00017834

[20] M. R. Olive , W. J. Peacock , E. S. Dennis , Nucleic Acids Res. 1991, 19, 7053. 1766868 10.1093/nar/19.25.7053 PMC332512

[21] F. U. Hoeren , R. Dolferus , Y. Wu , W. J. Peacock , E. S. Dennis , Genetics 1998, 149, 479. 9611167 10.1093/genetics/149.2.479 PMC1460183

[22] J. Bailey‐Serres , T. Fukao , D. J. Gibbs , M. J. Holdsworth , S. C. Lee , F. Licausi , P. Perata , L. A. Voesenek , J. T. van Dongen , Trends Plant Sci. 2012, 17, 129. 22280796 10.1016/j.tplants.2011.12.004

[23] H. Zhang , G. S. Demirer , H. Zhang , T. Ye , N. S. Goh , A. J. Aditham , F. J. Cunningham , C. Fan , M. P. Landry , Proc. Natl. Acad. Sci. USA 2019, 116, 7543. 30910954 10.1073/pnas.1818290116 PMC6462094

[24] H. Zhang , H. Zhang , G. S. Demirer , E. González‐Grandío , C. Fan , M. P. Landry , Nat. Protoc. 2020, 15, 3064. 32807907 10.1038/s41596-020-0370-0 PMC10493160

[25] H. Zhang , Y. Cao , D. Xu , N. S. Goh , G. S. Demirer , S. Cestellos‐Blanco , Y. Chen , M. P. Landry , P. Yang , Nano Lett. 2021, 21, 5859. 34152779 10.1021/acs.nanolett.1c01792 PMC10539026

[26] H. Zhang , N. S. Goh , J. W. Wang , R. L. Pinals , E. González‐Grandío , G. S. Demirer , S. Butrus , S. C. Fakra , A. Del Rio Flores , R. Zhai , B. Zhao , S. J. Park , M. P. Landry , Nat. Nanotechnol. 2022, 17, 197. 34811553 10.1038/s41565-021-01018-8 PMC10519342

[27] G. S. Demirer , H. Zhang , J. L. Matos , N. S. Goh , F. J. Cunningham , Y. Sung , R. Chang , A. J. Aditham , L. Chio , M. J. Cho , B. Staskawicz , M. P. Landry , Nat. Nanotechnol. 2019, 14, 456. 30804481 10.1038/s41565-019-0382-5 PMC10461892

[28] C. Y. Shi , E. R. Kingston , B. Kleaveland , D. H. Lin , M. W. Stubna , D. P. Bartel , Science 2020, 370, abc9546. 10.1126/science.abc9359 PMC8356967 33184237

[29] N. M. Hiers , T. Li , C. M. Traugot , M. Xie , Wiley Interdiscip. Rev. RNA 2024, 15, 1832. 10.1002/wrna.1832 PMC11098282 38448799

[30] F. Licausi , D. A. Weits , B. D. Pant , W. R. Scheible , P. Geigenberger , J. T. van Dongen , New Phytol. 2011, 190, 442. 20840511 10.1111/j.1469-8137.2010.03451.x

[31] C. Su , Z. Li , J. Cheng , L. Li , S. Zhong , L. Liu , Y. Zheng , B. Zheng , Dev. Cell 2017, 41, 527. 28586645 10.1016/j.devcel.2017.05.008

[32] T. Wang , Y. Zheng , Q. Tang , S. Zhong , W. Su , B. Zheng , J. Integr. Plant Biol. 2021, 63, 1475. 34020507 10.1111/jipb.13139

[33] Y. Zhao , S. Wang , W. Wu , L. Li , T. Jiang , B. Zheng , Nat. Commun. 2018, 9, 5011. 30479343 10.1038/s41467-018-07429-x PMC6258693

[34] S. P. Gordon , M. G. Heisler , G. V. Reddy , C. Ohno , P. Das , E. M. Meyerowitz , Development 2007, 134, 3539. 17827180 10.1242/dev.010298

[35] A. Suo , Z. Lan , C. Lu , Z. Zhao , D. Pu , X. Wu , B. Jiang , N. Zhou , H. Ding , D. Zhou , P. Liao , R. Sunkar , Y. Zheng , Genomics 2021, 113, 159. 33253793 10.1016/j.ygeno.2020.11.020

[36] L. Liu , S. Ren , J. Guo , Q. Wang , X. Zhang , P. Liao , S. Li , R. Sunkar , Y. Zheng , BMC Genomics 2018, 19, 111. 29764387 10.1186/s12864-018-4457-8 PMC5954288

[37] A. Kozomara , S. Griffiths‐Jones , Nucleic Acids Res. 2014, 42, D68. 24275495 10.1093/nar/gkt1181 PMC3965103

[38] S. F. Altschul , W. Gish , W. Miller , E. W. Myers , D. J. Lipman , J. Mol. Biol. 1990, 215, 403. 2231712 10.1016/S0022-2836(05)80360-2

[39] I. L. Hofacker , Nucleic Acids Res. 2003, 31, 3429. 12824340 10.1093/nar/gkg599 PMC169005

[40] M. J. Axtell , B. C. Meyers , Plant Cell 2018, 30, 272. 29343505 10.1105/tpc.17.00851 PMC5868703

[41] M. D. Robinson , D. J. McCarthy , G. K. Smyth , Bioinformatics 2010, 26, 139. 19910308 10.1093/bioinformatics/btp616 PMC2796818

[42] E. Varkonyi‐Gasic , R. Wu , M. Wood , E. F. Walton , R. P. Hellens , Plant Methods 2007, 3, 12. 17931426 10.1186/1746-4811-3-12 PMC2225395

[43] Y. Zhang , X. Zhang , Q. Tang , L. Li , T. Jiang , Y. Fang , H. Zhang , J. Zhai , G. Ren , B. Zheng , Sci. China Life Sci. 2024, 67, 1280. 38489006 10.1007/s11427-023-2466-7

