# Supplementary material for: Nanoparticle Delivery of Antisense miR162 Inhibits Invasive Habitat Adaption of Alternanthera Philoxeroides
Source: Adv Sci (Weinh). 2025 Mar 31;12(22):2416747. doi: 10.1002/advs.202416747 (PMC12165093; doi:10.1002/advs.202416747)
Supplement: Supplementary file 1 — Supporting Information [file ADVS-12-2416747-s002.docx]

Supporting Information

**Nanoparticle delivery of antisense miR162 inhibits invasive habitat adaption of *Alternanthera philoxeroides***

***Qianqian Hu*, *Erfeng Kou*, *Xiuzhen Liao*, *Ruiyi Qiu*, *Qi Tang*, *Huan Zhang*^*^, *Yun Zheng*^*^, *Ji Yang*^*^, *Binglian Zheng*^*^**

**
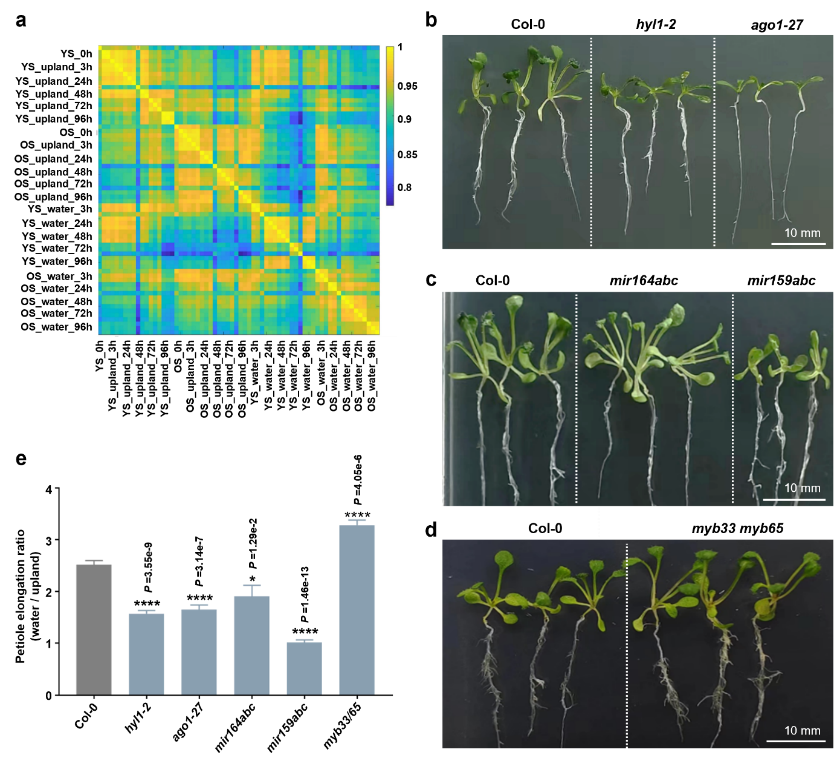
**

**Figure S1.** miRNA is required for submergence response in *Arabidopsis*.

a) Heatmap of the correlation coefficients between different samples and hierarchical clustering analysis. The x - axis and y - axis represent the same set of samples, with labels indicating the sample type (YS or OS), treatment (upland or water), and time point (0h, 3h, 24h, 48h, 72h, 96h). The legend on the right side of the figure from blue (low correlation, 0.8) to yellow (high correlation, 1.0), indicating the degree of correlation between different samples. YS: young stem, OS: old stem.

b-d) The growth phenotypes of *hyl1-2*, *ago1-27* (b), *mir164abc*, *mir159abc* (c) and *myb33myb65* (d) after submergence. Col-0 is wild type control.

e) Statistical analyses of the elongation ratio of petiole after submergence treatments. *****P*<0.0001 and **P*<0.05 were obtained by Student’s *t*-test.

**
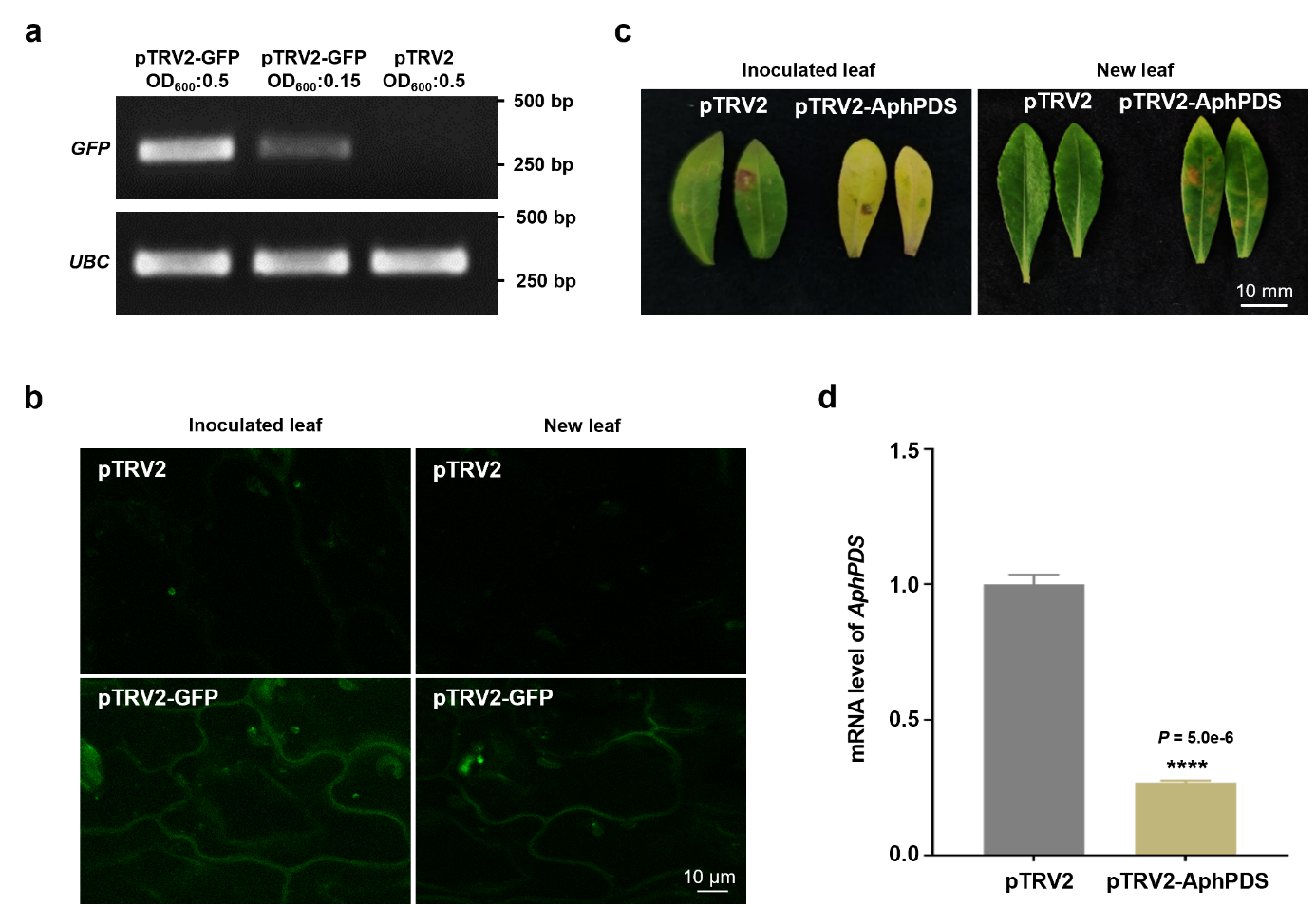
Figure S2.** TRV-based VIGS was successful in *A. philoxeroides*.

a) RT-PCR analyses to detect expression of *GFP* in leaves inoculated by pTRV2 and pTRV2-GFP. *AphUBC* was used as the loading control.

b) GFP fluorescence signals in leaf epidermal cells. pTRV2 as a negative control. scale bar = 10 µm.

c) *AphPDS* silencing using pTRV2-VIGS in *A. philoxeroides*. Scale bar = 10 mm.

d) qRT-PCR analyses showing expression of *AphPDS* in stems. Data are presented as mean ± standard errors (SE) from three biological replicates. *AphUBC* was used as an internal control. *****P*<0.0001 were obtained by Student’s *t*-test.

**
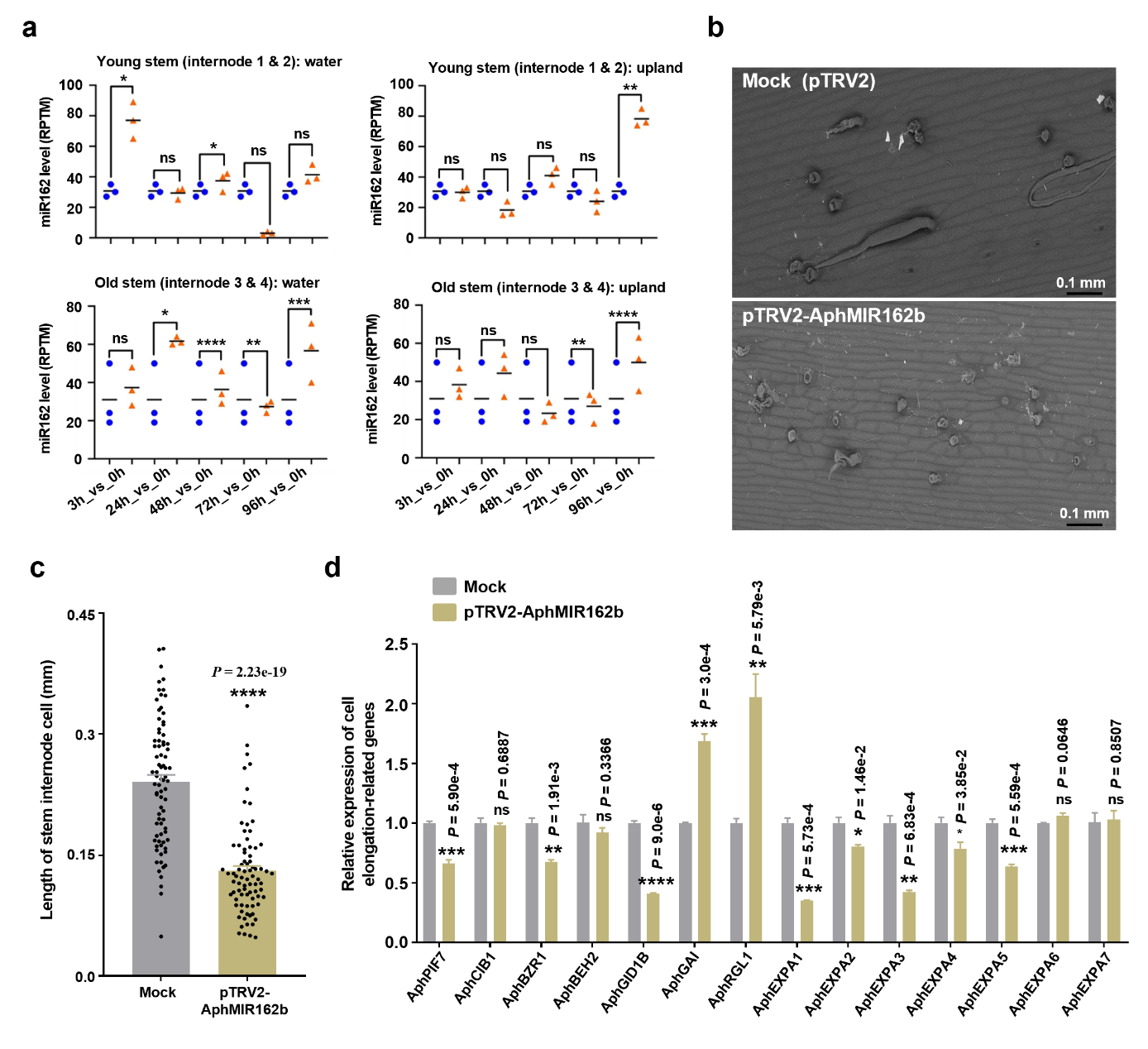
Figure S3.** miR162 reduction inhibits internode elongation in *A. philoxeroides*.

a) Box plots extracted from small RNA sequencing showing miR162 levels (RPTM) at different time points under water and upland conditions. The blue dots represent 0h and orange dots represent 3h, 24h, 48h, 72h and 96h, respectively.

b) Epidermal cells in the second internode of indicated genotypes observed with scanning electron microscope. Scale bars = 0.1 mm.

c) Statistical analyses of (b). Each dot represents one epidermal cell. **** *P* was obtained by Student’s *t*-test.

d) qRT-PCR analyses showing expression of light and hormone pathways genes in stems after knockdown of miR162. Data are presented as mean ± standard errors (SE) from three biological replicates. *AphUBC* was used as a control. *****P*<0.0001, ****P*<0.001, ***P*<0.01, and **P*<0.05 were obtained by Student’s *t*-test.

**
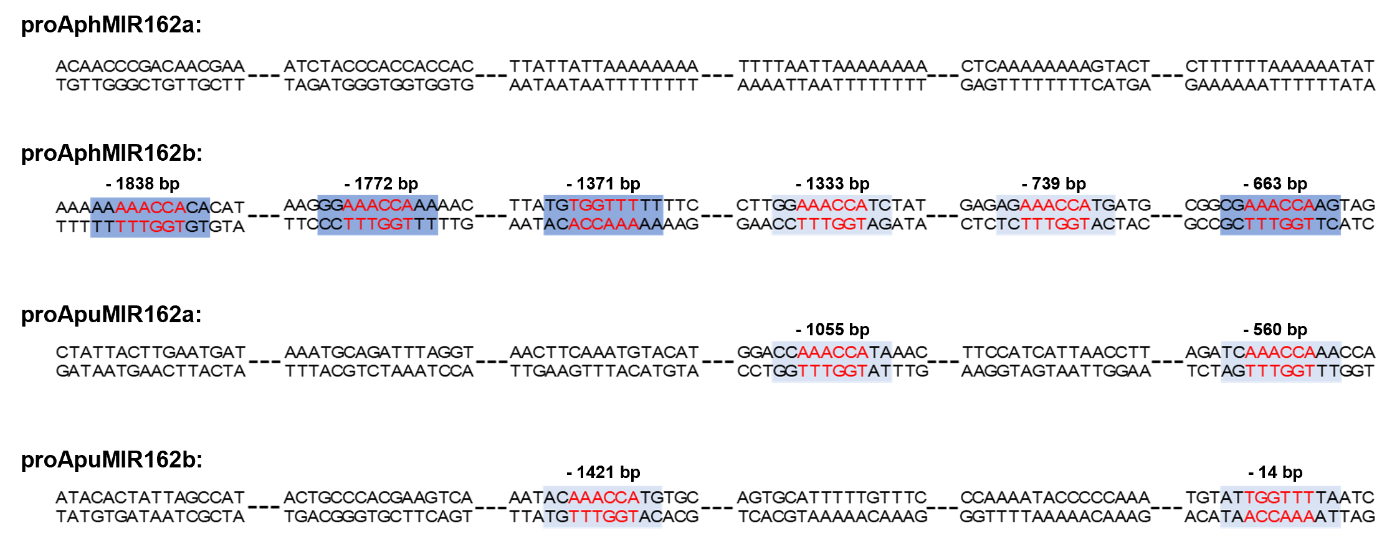
**

**Figure S4.** Distribution of AREs in the promoters of *MIR162* from *A. philoxeroides* and *A. pungens.*

2 kb sequences upstream of the transcription start sites (TSS) of MIR162 genes from *A. philoxeroides* and A. pungens are displayed. The core nucleotides are highlighted in red. The conserved ARE sequence (VVAAACCAVV), where "V" represents A, C, or G, is highlighted in dark blue. The degenerate ARE sequence (AAACCA), allowing for possible T in the flanking positions, is indicated by light blue shading.

**
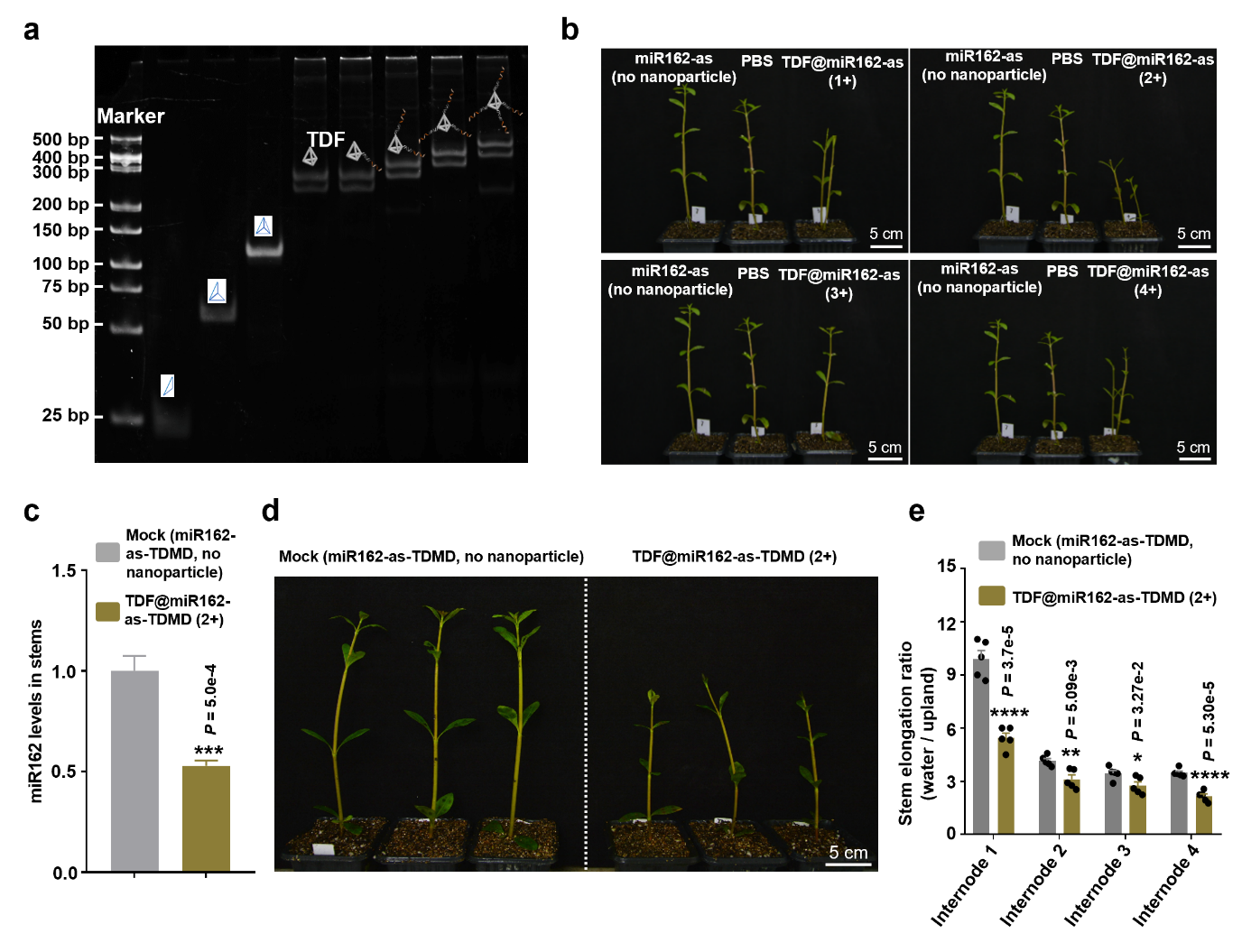
**

**Figure S5.** Effects of different nanoparticle-encapsulation strategies of TDF-miR162-as on submergence-induced stem elongation in *A. philoxeroides*.

a) 10% native PAGE showing the formation of the TDF and the miR162-as loaded TDF structures. Details in each lane (from left to right): marker; T13-a ssDNA; T13-a+T13-b; T13-a+T13-b+T13-c; TDF; TDF@miR162-as (1+); TDF@miR162-as (2+); TDF@miR162-as (3+); TDF@miR162-as (4+).

b) Plant height comparison among different types of TDF nanoparticle-delivery of antisense oligonucleotides of miR162 groups. Both miR162-as without nanoparticle and PBS buffer were used as the negative control. Scale bars = 5 cm.

c) RT-qPCR analyses showing the levels of miR162 in stems of *A. philoxeroides* inoculated by the TDF@miR162-asTDMD (2+). Data are presented as mean ± standard errors (SE) from three biological replicates. AphU6 was used as a control.

d) Growth phenotypes of water-logged *A. philoxeroides* after delivery by TDF nanoparticle-packaged as-miR162TDMD (TDF@miR162-asTDMD (2+)) and the mock control (miR162-asTDMD with no nanoparticle). Scale bars = 5 cm.

e) Statistical analyses of (d). Five plants were analyzed for each group. ****, ***, **, * indicate *P* < 0.0001, 0.001, 0.01, and 0.05. Student’s *t*-test was performed for (c, e).
